# Overcoming Glucocorticoid Resistance in Acute Lymphoblastic Leukemia: Repurposed Drugs Can Improve the Protocol

**DOI:** 10.3389/fonc.2021.617937

**Published:** 2021-03-11

**Authors:** Miguel Olivas-Aguirre, Liliana Torres-López, Igor Pottosin, Oxana Dobrovinskaya

**Affiliations:** Laboratory of Immunobiology and Ionic Transport Regulation, University Center for Biomedical Research, University of Colima, Colima, Mexico

**Keywords:** acute lymphoblastic leukemia, glucocorticoid-resistance, drug repositioning, signaling pathways, tamoxifen, cannabidiol, BH3 mimetics, tigecycline

## Abstract

Glucocorticoids (GCs) are a central component of multi-drug treatment protocols against T and B acute lymphoblastic leukemia (ALL), which are used intensively during the remission induction to rapidly eliminate the leukemic blasts. The primary response to GCs predicts the overall response to treatment and clinical outcome. In this review, we have critically analyzed the available data on the effects of GCs on sensitive and resistant leukemic cells, in order to reveal the mechanisms of GC resistance and how these mechanisms may determine a poor outcome in ALL. Apart of the GC resistance, associated with a decreased expression of receptors to GCs, there are several additional mechanisms, triggered by alterations of different signaling pathways, which cause the metabolic reprogramming, with an enhanced level of glycolysis and oxidative phosphorylation, apoptosis resistance, and multidrug resistance. Due to all this, the GC-resistant ALL show a poor sensitivity to conventional chemotherapeutic protocols. We propose pharmacological strategies that can trigger alternative intracellular pathways to revert or overcome GC resistance. Specifically, we focused our search on drugs, which are already approved for treatment of other diseases and demonstrated anti-ALL effects in experimental pre-clinical models. Among them are some “truly” re-purposed drugs, which have different targets in ALL as compared to other diseases: cannabidiol, which targets mitochondria and causes the mitochondrial permeability transition-driven necrosis, tamoxifen, which induces autophagy and cell death, and reverts GC resistance through the mechanisms independent of nuclear estrogen receptors (“off-target effects”), antibiotic tigecycline, which inhibits mitochondrial respiration, causing energy crisis and cell death, and some anthelmintic drugs. Additionally, we have listed compounds that show a classical mechanism of action in ALL but are not used still in treatment protocols: the BH3 mimetic venetoclax, which inhibits the anti-apoptotic protein Bcl-2, the hypomethylating agent 5-azacytidine, which restores the expression of the pro-apoptotic BIM, and compounds targeting the PI3K-Akt-mTOR axis. Accordingly, these drugs may be considered for the inclusion into chemotherapeutic protocols for GC-resistant ALL treatments.

## Introduction

Acute lymphoblastic leukemia (ALL) represents a heterogeneous group of hematological malignancies, originated from T (T-ALL) or B (B-ALL) cells progenitors. They suffered genetic alterations that preclude their further maturation and cause an unlimited self-renewal. Initially, malignant lymphocytes accumulate within the bone marrow (BM), where they constantly proliferate, displace healthy lymphoid precursors, devastate hematopoietic niches, and compromise the hematopoiesis. Later, a part of malignant cells leaves the BM and invades extramedullary sites, such as lymph nodes, spleen, liver, mediastinal space, and central nervous system (CNS). A proper treatment should begin immediately, otherwise clinical complications become incompatible with life (1, 2).

The established therapy consists of high-dose multi-agent protocols, which combine genotoxic drugs, antimetabolites, spindle inhibitors, and glucocorticoids (GCs). Albeit more than 80% of patients go to the remission after the induction therapy, there are also groups that are refractory to it. Many patients, who have reached the remission, will relapse later. A poor response to the initial GCs administration has been identified as a prognostic factor of unfavorable outcome (3, 4).

Over decades, synthetic GCs prednisolone (PRD), prednisone (PRED), and dexamethasone (DEX) were used widely as anti-inflammatory and immunosuppressive agents due to their lympholytic properties. They were among the first drugs, which were used for ALL treatment and remain as essential components of the antileukemic chemotherapy. The recent standard protocol, adopted by the Berlin-Frankfurt-Münster group, starts with 1 week of the GCs monotherapy, which serves as a prediction test and determines the future treatment strategy. Response to GCs varies among ALL patients, and GC resistance has been associated with an elevated risk of a minimal residual disease and poor survival (5–7). A more aggressive chemotherapy with toxic adverse effects is usually prescribed for these patients (3, 7–10). The understanding of underlying mechanisms could trigger the development of novel strategies that help to overcome steroid resistance in ALL.

Nowadays, much attention is paid not only to the development of new compounds, but also to a deeper understanding of the mechanisms of action of approved drugs, which may lead to their expanded or alternative use. Drug repurposing (or repositioning) is a very rational approach, since it implies the use of already approved drugs with identified mechanisms of toxicity and known side effects, thereby reducing the cost and time of the entire “from bench to bedside” process (11).

In the present review we have critically analyzed the available data regarding GC effects on leukemic cells, seeking the way to overcome the GC resistance by usage of certain repurposed drugs. The manuscript is divided into three parts. The first chapter describes the mechanisms of GC toxicity in sensitive cells. In the second chapter we discuss the mechanisms of GC resistance in ALL, with a focus on where the involved signaling pathways converge. In the third chapter we propose some drugs, already approved for treatments of other diseases, which can affect these converging points, thus overcoming/reverting GC resistance in ALL.

## Factors Determining GCs Effects In Lymphoid Cells

### Endogenous and Synthetic GCs

Primary endogenous GCs (cortisol in humans) are steroid hormones, generally produced by adrenal cortex in a response to physiological and/or emotional stress. GC synthesis is under the regulation of the hypothalamus-pituitary-adrenal axis. The duration of GC secretion is rather short, the clearance rate is rapid, and elevated GC levels, achieved during acute stress response, quickly return to their basal values. Because most cellular types in mammals express receptors for GCs (GRs), GCs display systemic effects, including a potent immunosuppression [reviewed in (12)]. According to early observations, adrenocorticotropic hormone administration leads to a decrease in mass of lymphoid organs in rats (13). Numerous subsequent studies demonstrated that GCs change the production of some interleukins, cytokines, and adhesion molecules, and cause cell death in lymphocytes (12).

Inverse relationship between the size of adrenal gland and thymus, the primary lymphoid organ, where T lymphocytes maturate, was also observed (14). At the same time, local GCs are naturally produced by stromal cells in the thymic cortex, providing the GC-rich microenvironment required for the T cells selection (15, 16). A crosstalk between the T cell receptor (TCR)- and GR- triggered pathways determines pro-survival or pro-apoptotic fates of thymocytes (17, 18).

Pharmacological effects of endogenous and synthetic GCs are similar. But synthetic GCs possess a greater relative potency and are significantly more stable [reviewed in (12)].

### Structural and Functional Diversity of GRs

GCs, being small lipophilic molecules, diffuse freely across the plasma membrane into target cells. Classically, they exert their effects by binding to their specific intracellular GRs, which are ligand-inducible transcriptional factors, belonging to the nuclear receptor superfamily. In the absence of a specific ligand, GRs are retained in the cytoplasm by their association with chaperone proteins. Ligand binding causes a formation of the GC-GR complex, its conformational change, and translocation to the nucleus, where it exerts genomic effects either through the direct binding to the specific DNA binding motif (the GC response element, GRE) or through the interaction with other transcriptional factors. GRE is an enhancer element, capable to modulate the activity of associated gene promoters, causing activation (transactivation) or inhibition (transrepression) of target genes expression [reviewed in (19, 20)]. Another, less appreciated regulatory function of GRs is related to the ability of GC-GR complexes to bind mRNA, triggering its rapid degradation (21, 22).

Although all GRs are encoded by unique *NR3C1* gene, their structure, stability, and functional characteristics are diverse. This diversity is generated by multilevel mechanisms at the transcriptional, post-transcriptional, translational, and post-translational levels [reviewed in (23–26)]. Based on these comprehensive reviews, here we briefly describe the mechanisms, relevant for GC resistance in ALL.

At the transcriptional level, there are several promoters that have alternative binding sites for various transcriptional factors that can increase or alternatively suppress the expression of the *NR3C1* gene (23). Among activators there are AP-1/AP-2, NF-κB, estrogen receptor (ER), cyclic-AMP responsive element binding protein (CREB), whereas GC responsive factor-1 and c-Ets-1/2 are reported as repressors. Interestingly, NF-κB also controls expression of anti-apoptotic and proliferative genes and it is frequently constitutively upregulated in ALL and may be related to drug resistance (27–29). AP-1 is involved in the GC response in ALL patients (30) and high CREB expression was correlated with a poor outcome (31).

Remarkably, *NR3C1* possesses binding sites for GRs themselves, providing an autoregulatory loop (23). Interactions of GRs with other relevant transcriptional factors can upregulate (interaction with c-Myb) or downregulate (interaction with c-Ets) the *NR3C1* expression (23). c-Myb was shown to interact with GR and enhances its expression level in pre-B-ALL (32, 33). Accordingly, a different tissue microenvironment and cellular context may contribute to the control of the *NR3C1* expression through upregulation of different transcriptional factors.

A different translation initiation of the GR transcript and an alternative RNA splicing result in a formation of several receptor isoforms, which possess different functional features (23–26).

Classical GRα protein is the most abundant isoform, accounting for about 90% of GR transcripts in all tissues (23). It efficiently binds GCs, possesses the nucleus-targeted sequence and DNA binding domain. Remarkably, there are eight alternative translation initiation sites in exon 2, resulting in eight GRα translational isoforms, named GRα-A to D, which are characterized by a different length of the N-terminal and by unique transcriptional target genes (34, 35).

Alternative splicing of the 9β instead of the 9α exon results in the GRβ isoform, which is unable to bind GCs, but is transcriptionally active (36). It resides constitutively in the nucleus and can alternatively regulate many genes, controlled by the GRα (37, 38).

GRγ isoform is less studied, but intriguing data evidencing unique GRγ properties were reported (39). GRγ is identical to GRα but contains an insertion of a single arginine near the nuclear localization signal, which slows down the nucleus-cytosol shuttling upon ligand binding when compared to GRα. GC and DNA binding capacities are similar to those of GRα, but their target genes are distinct. In particular, it was shown that GRγ controls nuclear genes, encoding mitochondrial proteins. GRγ is predominantly localized in the cytoplasm and in its unbound state targets mitochondria. The authors suggest unique functional profile of GRγ, which includes the regulation of mitochondrial function and ATP production.

Thus, distinct GR isoforms demonstrate non-redundant properties. Importantly, more than one isoform is usually found in the same cell, forming the cell-specific pattern. Consequently, cellular response to the GC application is the result of their complex crosstalk.

Stability of the GR mRNA is another factor, which may determine the GR expression level. mRNA stability is controlled by various mechanisms, including microRNAs (miRNAs) (23–26) and a previously mentioned GC-dependent mRNA decay (21, 22).

Further on, numerous GR mutations and polymorphisms may be related to either GC hypersensitivity or resistance [complete lists of GR mutations and polymorphisms known up to 2018 can be seen in (26)]. Finally, post-translational modifications, occurred at different physiological and pathological conditions, such as phosphorylation, ubiquitination, acetylation, nitrosylation or oxidation, are all capable to change the GR functional activity (23).

### Effects of GCs on Sensitive Lymphocytes

Effects of GCs on lymphoid cells include G1-phase cell cycle arrest and cell death, predominantly via the intrinsic (mitochondrial) apoptotic pathway (17, 20, 40–43).

To understand the early response of leukemic cells to GCs, parallel time-course metabolomics, proteomics and isotope-tracing studies were performed recently, using the B-ALL—derived cell line RS4;11 (44). The earliest genomic effect (4 h after the GC exposure) is a downregulation of the proto-oncogenic transcription factor *MYC*. CDK4, responsible for cell cycle progression, is decreased, whereas apoptotic markers *BCL2L11* (encoded BIM protein) and *CD93* are increased over time.

Puffal's group reported that DEX repressed the expression of genes, coding for key regulators of the early B cell development (*ITGA4, IL7R, BCL6*) as well as various genes related to the B cell receptor (BCR) signaling (*CD79B, CSK, FYN, BTK, PIK3CD, PIK3C2B, PIK3R2*). Pro-survival *BCL2* and *MYC* as well as *CXCR4* coding for the BM homing receptor were reported among the repressed, whereas pro-apoptotic *BCL2L11* and the major regulator of cellular redox signaling *TXNIP* among the activated genes. Remarkably, the mechanisms of cell death are most likely redundant, because no one among pro-apoptotic genes was determined as absolutely required (45).

IL7R and BCR pathways, in turn, work through the PI3Kδ stimulation, leading to the activation of ERK/MAPK and Akt/mTOR axes, involved in growth and survival (45, 46). Accordingly, PI3Kδ inhibition enhances the GC-regulated cell death even in resistant B-ALL (45). Different ALL were shown to be heterogeneous in the strength of the PI3K signaling [(47), and references therein].

Cell and tissue specificities of GC effects also depend on a specific pattern of the chromatin accessibility. Although the GR-associated transcriptome of lymphoid cells has not yet been decoded, lymphocyte-specific open (LSOs) and closed (LSCs) chromatin domains, characterized by different methylation degree, were described. The Bcl-2 family member *BCL2L11* was recently identified in highly accessible chromatin regions, critical for the GC-induced cell death in lymphocytes (48). BIM protein, which precludes the anti-apoptotic activity of Bcl-2, Bcl-XL, and Mcl-1, was demonstrated to trigger apoptosis in GC-sensitive cells (48, 49).

It is well-known that endogenous GCs are essential for regulation of energy metabolism in different human tissues under physiological and stress conditions (50). Expression of metabolism-related genes changed considerably in a response to GC treatments in ALL, causing strong alterations in cell metabolism (51–54). DEX treatment reduces the surface expression of the glucose transporter GLUT1, resulting in a decreased glucose uptake and a profound inhibition of glycolysis, both in cell lines and primary ALL (55). A consequent apoptotic cell death was correlated with the inhibition of glucose uptake (55). GLUT1 gene is not a direct target for GRs. The mechanism, underlying the inhibition of glycolysis, seems to be related to *MYC*, which is known to induce the expression of glucose transporters and some glycolytic enzymes in leukemic cells, and it is downregulated rapidly during GC treatments (44).

A switch to the mitochondrial oxidative phosphorylation (OXPHOS) for ATP production is a rescue strategy in GC-treated leukemic cells, in case of suppressed glycolysis (56, 57). When glycolysis is inhibited, mitochondrial activity appears to rely on autophagy (56, 58). There are various reports on a massive accumulation of autophagosomes in GC-treated ALL cells (44, 59, 60). Originally, autophagy was evolved as a pro-survival mechanism under starvation, but when the threshold level is exceeded, it can eventually lead to cell death (61). A non-protective autophagy was suggested to be an important process, preceding cell death in GC-treated leukemic cells (44, 59, 60).

**Figure 1:**
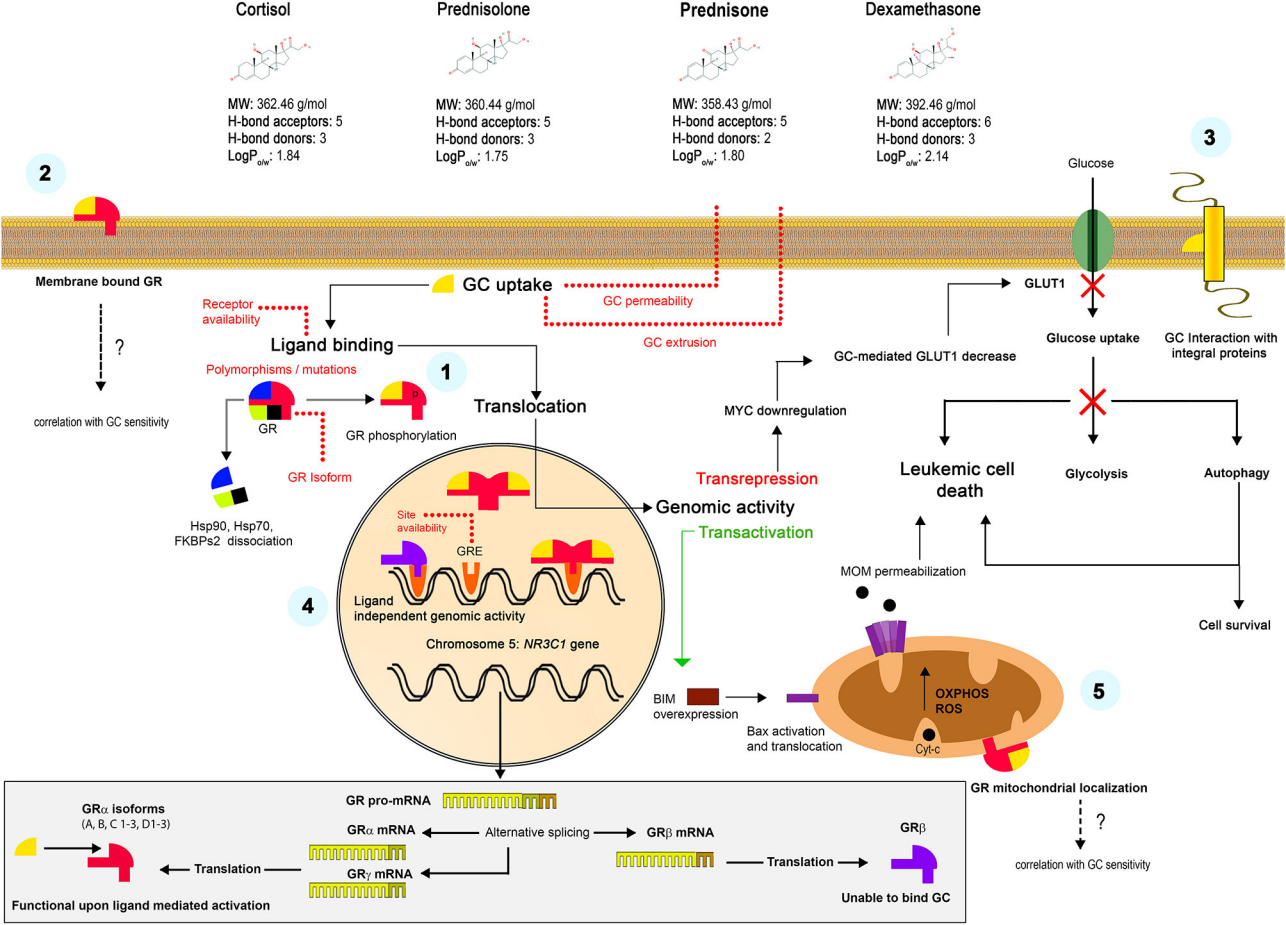
Mechanisms of the GC action. Liposoluble GCs freely diffuse through the plasma membrane. Classically, they bind to specific intracellular GRs (α or γ isoforms) with the formation of GC-GR complexes, their posterior translocation to the nucleus and interaction with the GRE, which results in a transactivation or transrepression genomic activity (1). Alternative non-genomic mechanisms were also proposed, including the interaction with surface receptors (2) or the GCs retention in the plasma membrane and the interaction with integral proteins (3). Unable to bind GC, but transcriptionally active β isoform constitutively resides in the nucleus and can alternatively regulate many genes (4). GC-GR complexes translocate to mitochondria and interact with the OMM proteins, causing non-genomic effects (5). The lower panel shows the formation of alternative GR isoforms. See Chapter 1 for more details.

Some rapid effects of GCs could be explained by non-genomic mechanisms. In particular, the translocation of the GC-GR complex to mitochondria instead of nucleus, with a subsequent direct interaction with the Bcl-2 superfamily proteins and triggering on the intrinsic apoptotic pathway was evidenced in experiments on mouse thymocytes (16, 62, 63). Another mechanism proposed the interaction with surface GRs and triggering of alternative signaling pathways (64). Finally, the accumulation of highly lipophilic GC molecules in plasma membrane was postulated, which alters the function of membrane integral proteins such as ion channels or receptors (65). Proposed mechanisms of the GC action in sensitive cells are summarized in the Figure 1.

## Mechanisms of GC Resistance in All

**Figure 2:**
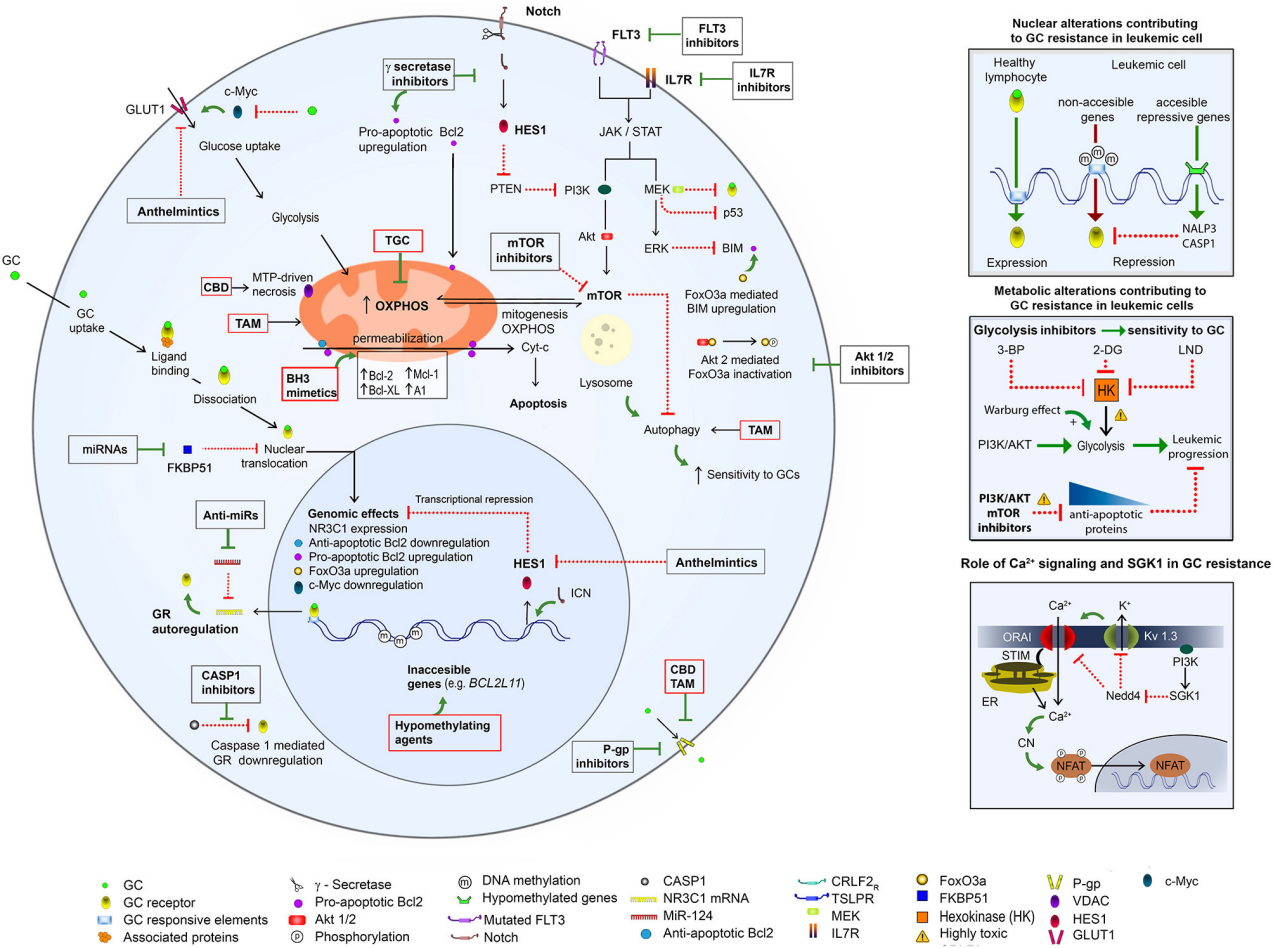
An overview of mechanisms of GC resistance in ALL and pharmacological strategies to overcome it. (**Left**) GC resistance in ALL is related to different genetic aberrations (see references in the text), which cause (1) upregulation of Notch, IL7R, Flt3, and MEK/ERK pathways, with a consequent upregulation of PI3K/Akt/mTOR and Glut1 and acceleration of cellular growth and metabolism; (2) downregulation of the proapoptotic proteins (BIM) and upregulation of the antiapoptotic proteins (Bcl-2, Bcl-XL, Mcl-1, and A1), with a consequent apoptosis inhibition; (3) overexpression of MDR proteins. A hypermethylation of *BCL2L11* results in its inaccessibility to the transcriptional upregulation by a GR (**left and upper right**). The mTOR activation causes upregulation of glycolysis (**middle right**) and OXPHOS, and inhibition of autophagy. Upregulation of glycolysis can be opposed by the inhibition of hexokinase (HK), the first glycolytic enzyme. Ca^2+^ signaling is involved in the NFAT activation via the Ca^2+^-dependent dephosphorylation by calcineurin (CN). A sustained Ca^2+^ signal is achieved due to a repression of the recycling of ORAI (main Ca^2+^ influx component) and Kv1.3 (mediating K^+^ efflux, which supports Ca^2+^ influx) proteins via the PI3K/SGK1 pathway (**lower right**). The above mechanisms can be opposed by inhibitors of FLT3, IL7R, γ secretase, Akt1/2, P-gp, glycolysis, PI3K/AKT, SGK, and mTOR as well as by BH-3 mimetics and hypomethylating agents. A possible toxicity of PI3R/AKT/mTOR inhibitors as well as of glycolysis/ hexokinase (HK) inhibitors 3-bromopyruvate (3-BP), 2-deoxy-D-glucose (2-DG), 1-(2,4-dichlorobenzyl)-1H-indazol-3 carboxylic acid) (lonidamine, LND) needs to be considered. Some re-purposed drugs may improve antileukemic protocols: (1) TGC, which targets mitochondria and OXPHOS; (2) CBD, which targets mitochondria, causes the MTP-driven necrosis and inhibits P-gp; (3) TAM, which targets mitochondria, induces autophagy, inhibits P-gp and enhances the sensitivity to GCs; (4) anthelmintics, which inhibit GLUT1 and Hes1. For more details please consult the text.

Here we present evidence for multiple mechanisms of GCs resistance (summarized graphically in the Figure 2). Most likely, different mechanisms may be responsible for GC resistance in the same leukemic clone.

### GC Resistance May Be Caused by an Altered GRs Expression

#### GRs Expression Is Heterogeneous in ALL

The level of the GRs expression among ALL patients and derived cell lines appears to be highly heterogeneous (Table 1). Sensitivity to GCs in hematological malignances was initially thought to be directly dependent on the number of functional GRs. This assumption seems to be logical and various reports confirmed it (66, 94–98), although conflicting results were also reported (7, 51, 71, 89). Regarding lineage differences, the levels of GRs were reported to be lower in T-ALL (90, 99–102), which is in line with the fact that T-ALL display GC resistance more frequently than B-ALL (6, 7). Additionally, a reduced binding affinity to DEX was revealed in T-ALL clinical samples as compared to B-ALL (102).

**Table 1:** GC sensitivity and GR expression in T- and B-ALL.

**ALL phenotype**	**Leukemic cells**	**Status/modifications**	**GC sensitivity**	**GR expression**	**Notch dependence**	**Components of resistance mechanism**	**References**
**T-ALL cell lines**						
CD3+ CD4+ CD8-	Jurkat parental (wt)	Relapse CD4+	Resistant	^*^	+	GRs↓Notch↑ PTEN↓ Akt↑nuclear GR translocation↓	(66–69)
	Jurkat /GR-A Jurkat /GR-B Jurkat /GR-C Jurkat /GR-D	Stable expression of GR translational isoforms in Jurkat (wt)	Sensitive Sensitive Sensitive Resistant	^****^ ^****^ ^****^ ^****^	ND	Reverted sensitivity to GCs Reverted sensitivity to GCs Reverted sensitivity to GCs Resistant, like Jurkat (wt)	(70)
CD3+ CD4+ CD8-	CCRF-CEM parental (wt)	Relapse	Sensitive	^***^	+	-	(68, 71–73)
	CEM-C7-14	Sub-clone of CCRF-CEM	Sensitive	^***^as parental	ND	-	(74, 75)
	CEM-C1-15	Sub-clone of CCRF-CEM, isolated without selective GC pressure	Resistant	^***^as parental	+	ND	(7, 73–75)
	CEM-C7//HDR	Prolong culturing CEM-C7–14 under hypoxia +single Dex treatment	Resistant	^*^/–	ND	GRs↓	(75)
	CEM-C7//H	Prolong culturing CEM-C7–14 under hypoxia (no Dex)	Sensitive	^****^	ND	-	(75)
	6T-CEM	HPRT-deficient	Sensitive	^***^	ND	-	(66)
	CCRF-CEM/MTX R3	Selected for resistance to MTX	Resistant	^**^	ND	GRs↓, nuclear GR translocation↓	(68, 72)
cyCD3+ CD4+ CD8+	MOLT-3	Relapse	Resistant	^*^	+	Notch↑ PTEN↓ Akt↑ GRs↓ nuclear GR translocation↓	(67, 76)
CD3+ CD4+ CD8+	CUTLL1	Relapse	Resistant	^**^	+	Notch/HES↑	(77, 78)
CD3+ CD4+ CD8+	UP-A t(8:14)(q24;q11) translocation LL13	Diagnosis	Sensitive	ND	-	-	(79)
CD3+ CD4+ CD8+	HBP-ALL	Diagnosis	Resistant	^***^	+	Notch↑	(66, 80), (81)
CD3+ CD4+ CD8+	T-ALL1	Relapse	Resistant	ND	+	Notch↑	(66, 77, 80)
CD3- CD4+ CD8+	ALL-SIL	Relapse	Sensitive	^**^	+	-	(7, 66, 80)
Precursor T lymphoblast	DND-41	ND	Sensitive	^**^	+	-	(76, 80)
	KOPTK-1	ND	Resistant	^*^	-	Akt↑ Notch↑	(76, 80)
ALL lymphoblasts	KE-37	Diagnosis	Sensitive	^****^	+	-	(68)
**B-ALL cell lines**							
Pro-B	HAL-01 CD3-	t (17, 19)(q22;p13) TCF3-HLF (E2A-HLF) fusion gene	Resistant	^*^	ND	Apoptosis resistance nuclear GR translocation↓	(68)
Pro-B	UOC-B1	Relapse TBL1XR1 delitions	Resistant	^****^	ND	Decreased GR recruitment at gene regulatory regionsnuclear GR translocation↓	(68, 82)
Pre-B	Reh parental (wt)	Relapse	Resistant	^*^/–	ND	GRs↓ BIM↓ p53↓ apoptosis resistance	(66, 68, 82)
	Reh/MEK4-KD	Reh wt MEK4 shRNA	Sensitive	^**^	ND	Reverted sensitivity to GCs: GR↑+ BIM↑	(82)
	Reh/MEK2-KD	Reh wt MEK2 shRNA	Sensitive	^*^/– as parental	ND	Reverted sensitivity to chemotherapy in general: pERK↓+ p53↑	(82)
Pre-B	NALM6 parental (wt)	Relapse	Sensitive	^****^	+	-	(66, 68, 83)
	NALM6/ DEX	NALM6 wt prolong exposure to DEX	Resistant	^*^	ND	GRs↓+ FLT3 point mutations	(84)
	NALM6/ HDR	NALM6 wt Prolong culturing under hypoxia + single Dex treatment	Resistant	ND	ND	Similar to CEM-C7//HDR?	(75)
	NALM6/ CELCR2-KO	NALM6 wt shCELSR2	Resistant	Lower than NALM6 (wt)	ND	Low ratio of BIM:BCL2 after PRED treatment	(85)
Pre-B	(697) parental (wt)	Relapse t(l;19) translocation	Sensitive	^***^	ND	-	(86)
	(697)/Bcl-2	Infected with recombinant Bcl-2 retrovirus	Resistant	^*^	ND	BCL-2↑+ GRs↓+ GSH↑	(87)
	(697)/DEX	(697): prolong exposure to DEX	Resistant	^*^	ND	GRs↓+ GSH↑	(84, 87)
Pre-B	RS4;11 Parental (wt)	Relapse	Sensitive	^*****^	ND	-	(84)
	RS4;11/DEX	RS4;11: prolong exposure to DEX	Resistant	^*^/–	ND	GRs↓+ FLT3↑ point mutations	(84)
Pre-B	(380) parental	Relapse Translocations: t (8, 14); t (14, 18)	Resistant	ND	ND	*IGH-BCL2* fusion *MYC-IGH* fusion Bcl-2↑+ Myc↑	(86, 88)
**Primary samples**						
Pediatric ALL patients(no phenotypes reported)	Xenografts	ND	Variable sensitivity depending on NR3C1 polymorphism	^**^	ND	BIM↓ in resistant samples	(68)
Pediatric ALL patients (no phenotypes reported)	*In vitro* primary samples	Relapse GR Somatic mutations not found in 49/50 patients	Variable sensitivity	Positive variable	ND		(89)
Pediatric ALL patients (no phenotypes reported)	Primary culture (BM/PB)	Diagnosis (43) Relapse (11)	Variable sensitivity	^***^	ND	Higher relation GRγ/GRα in resistant samples?	(90)
B-ALL	Freshly isolated (BM) primary cultures	Diagnosis	Resistant Sensitive	Variable	ND	Lower CELSR2 and higher BCL2 in GC resistant samples	(85)
Ph+-ALL	Xenograft derived strain (ALL-4CL)	Relapse	Resistant	^**^	ND	BIM ↓ Apoptosis resistance	(68)
CD11a+, CD19+;CD20+ CD38-; CD49e+	IM-9	ND	Sensitive	^*^	ND	-	(91)
Biphenotypic leukemias	Xenograft derived strain (ALL-7CL)	Diagnosis	Resistant	^****^	ND	BIM↓ Apoptosis resistance	(68)
9 ALL lymphoblasts	Primary cultures; patients exposed to PRD (n=49)	Relapse	Sensitive Resistant	^***^ ^*^	ND	Positive correlation between endogenous expression of NR3C1 in ALL cells and sensitivity to GCs and clinical outcomes	(66)
ALL lymphoblasts	Primary culture of patients exposed to PRD (n=9)	ND	Sensitive Resistant	^***^ ^*^	ND		(92)
T-ALL (incl. ETP) lymphoblasts	Primary culture from BM / Periphereal blood	Diagnosis	Resistant Variable depending on mutation	/– ^***^ (mutated)	ND	GRs↓ (deletion) Truncated GR, unable to bind to DNA [G (371)X mutation]	(93)
ETV6/RUNX1- ALL	BM	Relapse	Resistant	^*^/–	ND	Patients with *NR3C1* aberrations display a poor prognosis	(94)

* *Very low expression*;

** *Moderate expression*;

*** *Intermediate expression*;

**** *Increased expression*;

***** *Very high expression*.

*ND, Not determined*.

*HPRT, hypoxanthine phosphoribosyl transferase*.

*GSH, glutation*.

*MTX, methotrexate*.

Importantly, GCs by itself may cause an acute decrease in GR expression. This effect varied significantly, depending on the leukemic phenotype and on the chosen therapeutic protocol. The receptor re-establishment was observed predominantly during the first 15 days after the last DEX administration (90, 103). Remarkably, GC-resistant clones isolated from relapsed ALL patients usually express lower GRs levels due to alterations in *NR3C1* expression (66, 92, 94, 103–106). These data suggest that a chronic exposition to GCs in newly diagnosed ALL patients can promote the appearance and selection of ALL clones with a low GR expression, their evasion and re-appearance during the relapse.

However, high GR expression was found also in GC-resistant cases (Table 1). The opposite situation, when a high GC sensitivity is paralleled with a low level of GRs, is rare. Some early studies reported GC resistant clones, derived from sensitive cell lines, with apparently unaltered functional cytosolic GRs (74). These data argue for alternative mechanisms of the GC resistance, rather than simply to be caused by a decreased GR expression.

#### Somatic Loss-of-Function Mutations and Polymorphisms in the NR3C1 Gene May Alter GC Sensitivity

Somatic loss-of-function mutations in the *NR3C1* gene may change a proper functionality of GRs. Recurrent *NR3C1* inactivating aberrations, including deletions, missense, and nonsense mutations, which can be detected already at the first diagnosis, were reported to be responsible for GC resistance in pediatric T-ALL patients (93). Xiao et al. (98) reported frequent relapse-specific genetic alterations in adult patients with B-ALL, revealed by the longitudinal whole-exome sequencing analysis on diagnosis/ relapse pairs. In particular, recurrent truncated mutations were detected in the *NR3C1* gene (98). *NR3C1* deletions were also reported in ETV6/RUNX1-positive relapsed patients (94). Ectopic expression of the *NR3C1* reverses GC resistance, while the *NR3C1* deletion, in contrast, confers a resistance to GCs in ALL cell lines and xenograft models (66). Polymorphisms were found in healthy individuals and ALL patients. Moreover, it has been observed that GR polymorphisms conferred an increased or decreased GC sensitivity (107).

#### Epigenetic Regulation of *NR3C1* Expression May Be Altered in ALL

Alterations in the GR protein expression, associated with the methylation status of the *NR3C1* gene, have been described in some human pathologies [reviewed in (108)], but not in ALL. However, there are other epigenetic mechanisms regulating the *NR3C1* expression, such as silencing or repressive RNA. ALL patients exhibit high levels of miRNAs. In particular, miR-124 is overexpressed in GC resistant leukemic cell lines and poor PRD responders. *NR3C1* was found to be a target for miR-124, which acts as a GR suppressor, inhibiting the apoptosis induced by DEX (109). Conversely, *FKBP51*, a GR repressor that decreases GR autoregulation and activity, was shown to be a target for miR-100 and miR-99a. miRNA expression, which limits the *FKBP51*, reestablishes the *NR3C1* autoregulation and activity and confers GC sensitivity (110).

#### Differential Expression of GR Isoforms in ALL

Since GR isoforms are functionally non-redundant (see section Structural and Functional Diversity of GRs), their pattern in leukemic cells may be associated with a different GC sensitivity. In particular, GC resistance was associated with a high GR β/α ratio (111–113) that may be explained by the fact that GRβ alternatively regulates GRα-dependent genes (discussed in Structural and Functional Diversity of GRs). Pro-inflammatory cytokines TNFα and IL-1 can selectively upregulate the GRβ expression, as it was demonstrated for leukemic cell lines (112). Remarkably, leukemic niches in B-ALL are characterized by a proinflammatory microenvironment, producing enhanced levels TNFα, IL-1 and IL-12 (114) that may support the GC resistant phenotype.

GRγ is an important positive regulator of mitochondrial function (see section Structural and Functional Diversity of GRs). GRγ up-regulation is related to an increase in the mitochondrial mass, oxygen consumption, and ATP production (39). Accordingly, an enhanced GRγ expression is associated with some GC resistant cases (90, 115).

As far as different GR translational isoforms can mediate differential regulatory patterns of GC-induced genes, the question about their capacity to induce apoptosis in ALL was addressed. With genetically modified Jurkat cells, expressing individual GR isoforms GRα-A-D, it was demonstrated that DEX efficiently decreased Myc expression and induced apoptosis in GRα-A-B but not in GRα-D expressing cells (70).

#### GR May Be Cleaved by Upregulated Caspase 1

A low somatic methylation of the *CASP1* gene and its activator *NLRP3* was observed in ALL patients, with a resulting upregulation of caspase 1. It was revealed that GR may serve as a target for inflammasome and may be cleaved by caspase 1, resulting in a decreased receptors' number (116).

### Alterations in Signaling Pathways in ALL May Be Involved in GC Resistance

Genetic alterations, which cause ALL, occur in two steps. Chromosomal rearrangements, which result in upregulation of oncogenic proteins and maturation arrest, are considered as driving leukemogenic events and are associated with unique expression profile. Gene rearrangements in ALL often place the oncogenic transcriptional factors under the control of promoters or enhancers of the BCR/TCR or *BCL11B* genes, among others. During the pre-leukemic phase additional mutations occur and give rise to ALL. These secondary mutations alter basic cellular processes, including survival, cell cycle progression, proliferation, and apoptosis. They are related to a variety of signaling pathways, including Notch, Il7R/JAK/STAT, RAS/MEK/ERK, and PTEN/PI3K/AKT/mTOR ones. Several comprehensive reviews, which describe in detail sequential genetic rearrangements and mutations in leukemogenesis were published recently (117–120). Targeting mutated genes and pathways was proposed as a basis for the “precision medicine” (121, 122). However, this strategy requires further studies, concerning a complex crosstalk between altered signaling pathways, to reveal the most promising therapeutic targets, in particular when patients present multiple genomic lesions (120). In addition, whereas genetic biomarkers are widely used for risk predictions in B-ALL, few genetic abnormalities were reported to show a prognostic significance in T-ALL (119, 123). Accordingly, functional studies should be of a primary importance. In a continuation we will discuss those signaling pathways, which are upregulated in GC resistant phenotypes, and intend to determine the most frequent abnormalities and convergent points.

#### Notch Activation

More than 50% of human T-ALL are known to exhibit Notch activating mutations (80, 119, 124). An enhanced expression of Notch receptors was also reported in B-ALL primary samples and cell lines (125). Remarkably, an aberrant Notch upregulation is associated not only with an increased proliferation but also with chemoresistance. Notch inhibition by a highly potent γ secretase inhibitor (GSI) reversed chemo- and GC- resistance in both B- and T-ALL [(77, 125); Table 1].

Notch is involved in the regulation of the *NR3C1* expression and GR protein levels. The underlying mechanism was shown to involve *HES1*, a transcriptional repressor, which is upregulated by Notch signaling and binds to *NR3C1* promoters, responsible for the GR autoregulation (67, 77, 126). Notch-dependent positive regulation of mTOR pathway in ALL is also related to *HES1* (127). *HES1* inhibits the tumor suppressor phosphatase and tensin homolog (PTEN), which is a negative regulator of the phosphatidylinositol 3-kinase (PI3K), whereas the activation of PI3K is the primary step in the PI3K–AKT–mTOR1 axis (128). Notch acts as a positive modulator of the interleukin 7 (IL-7) receptor, IL7R (129).

#### Upregulation of Cytokines' Receptors in GC Resistance

IL-7 is a cytokine, produced by thymic and BM stroma, which supports survival and proliferation of both healthy and leukemic lymphocytes. Activating mutations in the *IL7R*α gene was reported in 6% of pediatric ALL, with a higher prevalence in T-ALL (130, 131). Primary T-ALL samples developed GC resistance, when cultured with IL-7 (123). GCs induce their own resistance by activating the IL7R (123). IL7R mediates its downstream effects through the JAK/STAT and PI3K/Akt/mTOR pathways. Deprivation of IL-7 or blockade of downstream effectors enhances the efficiency of DEX in T-ALL cells (132, 133). Whole genome and targeted exome sequencing, undertaken recently in T-ALL patients, revealed frequent (32%) IL7R mutations among the abnormalities, identified for 151 genes (134). Specific IL7R mutation, when expressed in steroid-sensitive cell lines, induces GC resistance through an upregulation of MEK-ERK and PI3K/Akt/mTOR. Accordingly, IL7R inhibitors revert the apoptosis development in response to GCs (134). Inhibitors of MEK and PI3K/Akt efficiently block the IL7R signaling (135).

The cytokine fms-like tyrosine kinase 3 ligand (FL) and its receptor FLT3 form an important axis in the hematopoiesis regulation. An aberrant up-regulation of FLT3 is commonly found in ALL, including a high intrinsic FLT3 level or gain-of-function mutations that promote constitutive FLT3 activity (136). Similar to IL-7, the FLT3 signaling converges with the PI3K/Akt/mTOR pathway. Some GC resistant ALL cells are characterized by a constitutive activation of the FLT3 signaling. They also exhibit a suppressed GR activity due to the Akt-mediated phosphorylation (76, 84).

#### MAPK Axis

Genome-scale short hairpin RNA screening was used to identify the mediators of GC resistance in B-ALL cell lines (137). Two different mitogen-activated protein kinases (MAPK), MEK2 and MEK4, were shown to be important for GC resistance but act through distinct mechanisms. MEK4 knockdown (KD) significantly increases both GR expression and transcriptional activity. The latter phenomenon seems to be related to the phosphorylation of GR on Ser226, which may cause its nuclear export and degradation. Accordantly, PRD-induced expression levels of *GILZ* and *BIM*, related to apoptosis, are higher in MEK4 KD samples. In contrast, MEK2 KD does not affect the GR expression but increases the sensitivity to various cytotoxic agents. Underlying mechanism involve the MEK2-dependent ERK suppression, which in turn causes an upregulation of the p53 and sensitizes leukemic cells to a drug-induced apoptosis (137).

#### Metabolic Re-programming and Upregulation of the PI3K/Akt/mTOR Pathway Is Related to GC Resistance in ALL

As for other cancer types, a re-programmed energy metabolism is typical for ALL. It includes the upregulation of both glycolysis and OXPHOS. ATP production predominantly via glycolysis (Warburg effect) gives the advantage to use the truncated tricarboxylic acid (TCA) cycle for biosynthesis of lipids, proteins, and nucleic acids (57, 138). The expression pattern of genes, associated with the glucose metabolism, is different in GC-sensitive and GC-resistant B-ALL. In particular, expression levels of hypoxia-inducible factor-1 alpha (HIF-1α), glucose transporters, carbonic anhydrase 4 (CA4), and glyceraldehyde-3-phosphate dehydrogenase (GAPDH) are significantly higher in GC-resistant ALL (52, 139). An enhanced glucose consumption and glycolytic rate are correlated with GC resistance in ALL. Inhibition of glycolysis, either by RNA of interference or by synthetic compounds, reverts GC resistance in cell lines and primary samples of both B- and T-ALL (52, 54). Inhibitors of glycolysis and OXPHOS pathways were shown to enhance the sensitivity to GCs in T-ALL *in vitro* (54). A synergy between GCs and metabolic inhibitors was suggested as a valuable strategy for ALL treatments (56).

Under conditions of an increased rate of glycolysis and, as a consequence, limited availability of pyruvate, the TCA cycle is replenished with glutamine, which also leads to an increase in the rate of glutaminolysis in most types of cancer (57, 138, 140). Leukemic cells require a rapid source of ATP and, at the same time, enough biosynthetic precursors, for their accelerated proliferation. Consequently, ALL, especially GC-resistant ones, predominantly make ATP via glycolysis (54), whereas glutaminolysis serves as an extra source of biosynthetic precursors (57, 140). In Notch1-induced T-ALL, glutaminolysis represents a key carbon source and is critically dependent on the up-regulation of mTOR pathway (127, 141, 142). This metabolic reprogramming was shown to induce resistance to anti-Notch1 therapy (141). Consequently, the inhibition of glutaminolysis and mTOR was proposed as a potential strategy against Notch1-driven and, even, against anti-Notch1 therapy resistant ALL (141, 142). GCs not only suppress glycolysis, but also prevent the entry of glutamine into TCA cycle (44). It remains to be elucidated, whether the suppression of glutaminolysis by GCs in GC-resistant ALL is insufficient to minimize its metabolic contribution.

A balance between OXPHOS and glycolysis is under the control of the outer mitochondrial voltage-dependent anion channel, VDAC1, which mediates ion and metabolite exchange between mitochondria and cytosol (143). A comparison of GC-resistant and GC-sensitive B-ALL lines revealed that an enhanced VDAC1 expression is a crucial biomarker for GC-resistance (144). In addition to its role in the metabolic reprogramming, the VDAC1 closed or open conformation favors ALL death or survival, respectively (57).

PI3K-Akt pathway is constitutively hyperactivated in more than 80% of primary T- and B-ALL (45, 47, 145–149). Mutations in *PIC3CA* and *PIKRA*, encoding catalytic and regulatory PI3K subunits, are observed frequently in different ALL subtypes (150). Akt up-regulation is required for an increased glucose metabolism, which underlies a sustained cell growth (126, 151). Akt up-regulation is characteristic for GC-resistant phenotypes (152). Akt phosphorylates GR at Ser134, which prevents its translocation from the cytosol to the nucleus (76). In T-ALL, the inhibition of Akt2 enhances the sensitivity to GCs more efficiently than the inhibition of Akt1 (152).

Critical downstream effector of the Akt is the mammalian target of rapamycin (mTOR), which is upregulated in many cancers (153, 154). mTOR contributes to leukemogenesis and GC resistance in ALL (155). PI3K/Akt/mTOR pathway appears to be critical for a proliferative response of leukemic cells to CXCL12, IL-7 and different stroma-derived mediators (156). Notch1 and Akt pathways interplay in ALL through *HES1*, which negatively controls *PTEN*, the main negative regulator of Akt signaling [(126, 151), see also section Notch Activation]. Overall, T-ALL patients often display an increased PI3K/Akt/mTOR pathway activation (145, 157). mTOR is known as an important regulator of a balance between survival, autophagy, and cell death (153, 154). The underlying mechanism to a large extent is related to mTOR involvement into the regulation of mitochondrial function and biogenesis [(158) and references therein].

#### Autophagy May Be Involved in GC Response

Autophagy is an essential recycling process, which is responsible for degradation of unnecessary, dysfunctional or damaged organelles and proteins in living cells. mTOR is a central checkpoint that negatively regulates autophagy. Metabolic stress is known to cause autophagy (61). In the context of anti-cancer treatments, autophagy may allow cells to survive during chemotherapy but may also act as a pro-death mechanism. This dual outcome is reported for various types of cancer (159, 160), including acute leukemias of myeloid and lymphoid lineages (161).

During unfavorable metabolic circumstances, caused by chemotherapy, autophagy may provide energy and macromolecules, required for survival and proliferation of cancer cells. Autophagy is an important mechanism, which maintains OXPHOS in leukemic cells, when glycolysis is inhibited by GCs (see section Effects of GCs on Sensitive Lymphocytes). As a result, GC-treated cells may be more sensitive to mitochondria-targeted compounds. A combination of these two classes of drugs was shown to cause a synergistic effect (56).

The expression of autophagy-associated genes was studied in samples, derived from B-ALL pediatric patients, where a differential expression was demonstrated for the GC-sensitive group as compared to the GC-resistant one (162). In general, key autophagy inducer genes are downregulated, while the inhibitors of autophagy are upregulated in GC-resistant cells (162). Activation of *BECN1*, a key autophagy inducer, is required for the DEX-dependent cell death in ALL (42) and for a sensitization of DEX-resistant ALL cells to obatoclax (163, 164) and MEK1/2 inhibitor (165).

In GC-resistant, in contrast to GC-sensitive ALL cell lines, autophagy is not induced by DEX (60, 164). Interestingly, a sensitization to GCs is achieved in GC-resistant Jurkat cells by a co-treatment with the autophagy-inducing drug tamoxifen (TAM) (166). Obatoclax reverts the GC resistance through the autophagy-dependent necroptosis, while knock-down of the autophagy-related gene 7 (*ATG7*) and *BECN1* completely prevents the re-sensitization to DEX (163, 164). These data indicate that autophagy can contribute to death of GC-treated cells.

#### Hypoxic Conditions Favor the GC Resistant Phenotype

BM leukemic niches represent a sanctuary for blasts, which therefore evade chemotherapy and are responsible later for a relapse. Like hematopoietic niches, they possess a hypoxic microenvironment (167). Leukemic cells, cultured under hypoxic conditions *in vitro*, were shown to develop the GC resistance (75, 168). Hypoxia is a signal, regulated mainly by the HIF-1α. Under hypoxic conditions, T-ALL cells up-regulate the HIF-1α expression, which activates Notch1 signaling, favoring cell cycle progression and limiting GC sensitivity [(67, 77, 169); discussed in Notch Activation]. Under hypoxic conditions, HIF-1α is overexpressed and ALL cells response to PRED is impaired, as evidenced by lower levels of BIM and higher levels of antiapoptotic proteins Mcl-1 and Bcl-2 (168). Therefore, hypoxia, together with a high production of IL-7 and Notch ligands (see Notch Activation and Upregulation of Cytokines' Receptors in GC Resistance), form a complex microenviromental network in leukemic niches, favorable for the maintenance of GC resistant clones. GC-resistant ALL cell lines, derived from relapsed cases, show mostly a low GR level (Table 1).

#### Resistance to GCs Can Be Mediated by Ion Channels and Ca^2+^ Signaling: The Role of SGK1

Ca^2+^ signaling is a principal component in the activation of healthy lymphocytes via TCR/BCR and the expression of about ¾ of genes, involved in the activation, is Ca^2+^-dependent (170). ALL cells proliferation does not depend on the antigen binding to TCR or BCR, but still relies on the otherwise altered Ca^2+^ signaling. There is also an invariant signaling axis for the proliferation of both healthy lymphocytes and ALL cells, including a sustained Ca^2+^ influx via the plasma membrane Ca^2+^ channel, CRAC, Ca^2+^-binding protein calmodulin, which activates calcineurin; the latter dephosphorylates the NFAT, allowing its import by nucleus and a consequent initiation of genes transcription [Figure 2; for a review see (170, 171)].

GCs acutely induce the expression of serum-and-glucocorticoid-inducible kinase-1 (SGK1), which is involved in a variety of pathologies, including tumor growth and resistance to GC-chemotherapy [for a review see (172, 173)]. In particular, SGK1 was found among key upregulated genes in GC-resistant B-ALL (162). Among multiple SKG1 targets is the Orai-1, the main channel-forming subunit of CRAC. SGK1 phosphorylates the Nedd4-2 protein, which binds then the 14-3-3 protein. The resulting protein complex is unable to ubiquinate the Orai-1 protein, thus precluding its degradation (174). An enhanced CRAC activity underlies pro-survival scenarios in tumor cells (174, 175). Activation of CRAC by thapsigargin suppresses, whereas chelation of intracellular Ca^2+^ potentiates, the sensitivity of ALL to the GC treatment (176).

CRAC is not unique route for Ca^2+^ entry. TRPV5 and TRPV6 channels, which display a high Ca^2+^/Na^+^ selectivity, are scarcely expressed in quiescent healthy T cells, but robustly in T-ALL (177, 178). SGK1 activity increases the membrane surface expression of TRPV5 and TRPV6 channels (174, 179). Another important member of the TRP channels family, TRPC3, is less selective albeit permeable for Ca^2+^. In T-ALL upon the mitogenic stimulation it can mediate an extra Ca^2+^ signal, additional to the CRAC-generated one (180). Notably, TPC3 gene expression is strongly upregulated upon T-cells activation (177). A specific block of the TRPC3 by Pyr3 suppresses the GC-induced Ca^2+^ signal in ALL and synergistically enhances the DEX-mediated cell death (176). K^+^ efflux via K^+^-selective channels causes membrane repolarization, which underlies a sustained Ca^2+^ entry via CRAC [Figure 2; (170, 171)]. Voltage-dependent K^+^ channels in B and T cells are functionally represented by the single member, Kv1.3 (181, 182). The Kv1.3 current is robustly presented in T-ALL, albeit it is lacking in B-ALL (183, 184). The surface expression of Kv1.3 channels is downregulated by Nedd4-2 and upregulated by different SGK isoforms (185). Therefore, it may be hypothesized that an increase in the Kv1.3-mediated current by SGK1 may contribute to the GC-resistance in T-ALL but not in B-ALL, via a promotion of Ca^2+^ entry. In several malignant tumors, including ALL, there is an aberrant expression of the cardiac K^+^ channel hERG, which in its non-conducting (closed) state forms the signaling complex with β-integrin and CRC4. This aberrant signaling complex mediates both ERK1/2 and PI3K/Akt pro-survival pathways, which cause the SKG1 induction. Consequently, hERG1 contributes to the GC resistance in B-ALL, whereas a pharmacological block of hERG1 sensitizes B-ALL to GC treatments (186). Development of low molecular weight inhibitors with a high (100-fold) preference for SGK1 as compared to the generically similar kinase Akt and preclinical tests on colorectal cancer supports a synergistic effect of the SGK1 inhibitors with radio- and chemotherapy (173). At the same time, SGK1 can increase the degradation and ubiquitylation of Notch protein (187) and Notch pathway is up-regulated in most patients with T-ALL [reviewed in (171)].

### Alterations in the Regulation of Apoptosis Are Related to GC Resistance

#### Bcl-2 Superfamily

The GC-induced cell death in sensitive leukemic cells is executed mainly through the intrinsic apoptotic pathway (discussed in section Effects of GCs on Sensitive Lymphocytes). Specific pattern and interactions of pro- and anti-apoptotic proteins of the Bcl-2 family determines the sensitivity to the apoptosis in leukemias (57).

The pro-apoptotic BIM, belonging to the BH3-only group, is the most studied in ALL. As it was mentioned previously, GC administration causes BIM overexpression in sensitive cells (68, 188), since GCs bind and stimulate the promoter, situated in lymphocyte-specific open chromatin domains (48, 49). In contrast, BIM enhancer was found to be highly methylated and therefore inaccessible for transcription in the GC resistant ALL (48).

Transcription factor FoxO3a is a well-known BIM regulator, which binds to BIM promoter and enhances BIM expression in a sensitive phenotype (152, 189). Akt2 kinase, up-regulated in the GC-resistant ALL, is responsible for Fox3a phosphorylation at Ser253. Resulting p-FoxO3a (Ser253) form is unable to translocate to the nucleus. Akt2 possesses a stronger binding capacity to FoxO3a than Akt1. Akt2 silencing significantly decreases FoxO3a phosphorylation at Ser253 and Akt2 inhibitors efficiently restore the GC resistance in ALL. DEX administration can upregulate the FoxO3a expression and decrease the p-FoxO3a, as a result favoring BIM expression and apoptosis (152).

In T-ALL, the inactivation of Notch signaling as well as limitation of the PI3K/mTOR pathway leads to a decreased Akt expression and activity, thus, promoting FoxO3a nuclear translocation and upregulation of BIM expression (77, 190).

Mutations of genes, which activate the extracellular signal-regulated kinase (ERK) pathway, are recurrently found in ALL. It has been also observed that BIM protein can be phosphorylated by ERK at Ser55, Ser65, and Ser100, preventing its efficient interaction with BAX, and, consequently, impeding apoptosis (191, 192).

ALL cells usually display high levels of anti-apoptotic proteins [reviewed in (35)]. In particular, a high level of Mcl-1 expression was associated with the resistance to PRED in MLL-rearranged infantile ALL clinical samples (193). Downregulation of Mcl-1 by RNA of interference induces PRED sensitivity in an ALL cell line (193). A comparative analysis of gene expression in clinical samples, obtained from children diagnosed with B-ALL, reveales an upregulation of the pro-survival Bcl-2 family members Mcl-1 and Bcl-2A1 (A1) in GC-resistant samples (162). Overexpression of Bcl-2 as well as of Mcl-1 tends to protect against the GC-induced apoptosis *in vitro* (139, 194). It was suggested that upregulation of Mcl-1 is related to upregulation of Akt/mTOR pathway (127). In this context, mTOR inhibitor rapamycin causes Mcl-1 downregulation and sensitizes ALL cells to GCs (188). Similarly, PI3K/mTOR inhibitor BEZ235 decreases the levels of the pro-survival Bcl-2 members but increases that of BIM (190).

#### p53 and MDM2

Mutations that inactivate p53, a genome-guardian protein, responsible for genetic stability and DNA repair, are frequently observed in several cancer types. The murine double minute 2 (MDM2) protein represents the main negative regulator of the p53 activity. MDM2 overexpression has been found in BM samples from ALL patients. Interestingly, p53 expression in MDM2 overexpressing patients is poorly detected, which correlates with an unfavorable outcome (195). In 11 different T- and B-ALL cell lines high levels of MDM2 are detected. Also, the analysis of 42 B-ALL relapsed patients demonstrated that most of them possess MDM2 alterations. Those, who failed to re-induce a remission after the chemotherapy with the use of PRED, were characterized with a high level of MDM2 expression (196, 197). MDM2 contribution to GC resistance was also evidenced in a preclinical ALL model. Mixed lineage leukemic xenografts in deficient mice were treated with DEX and, additionally, with RG7112, a MDM2 inhibitor. Mice, treated with RG7112, display p53 overexpression and cell cycle arrest, while DEX efficiency to induce apoptosis is increased (198). It has been reported that GR can interact with p53 and, upon GC administration, MDM2 was recruited, promoting a degradation of both GR and p53 (199).

### Multidrug Resistance Contributes to GC Resistance

Other aspect that might explain the lack of sensitivity to GCs is a higher expression of drug-efflux pumps or transporters such as P-glycoprotein (P-gp), multidrug resistance 1 (MDR1) pump, and multidrug resistance-associated protein, MRP1 (200). Although P-gp is overexpressed both in GC- resistant and sensitive pre-B ALL cells, its activity does not correlate with GC sensitivity (87, 201). As it was demonstrated recently, inhibition of an upregulated MDR1 in a GC-resistant B-ALL sensitized cells to DEX (202).

### Integrative Genomic Analysis as a Tool to Reveal Key Elements in GC Resistance

Many pathways are involved in GC resistance in ALL (see sections GC Resistance May Be Caused by an Altered GRs Expression, Alterations in Signaling Pathways in ALL May Be Involved in GC Resistance, Alterations in the Regulation of Apoptosis Are Related to GC Resistance, and Multidrug Resistance Contributes to GC Resistance). The challenge is to reveal how these different pathways interact, to determine the exact position of each component and key elements in a complex signaling.

Functional genomics studies and a genome-wide shRNA screen, performed by Pufall's group, have identified two classes of GC-regulated genes, which contribute to GC sensitivity in B-ALL: (a) effector genes, which contribute to cell death and (b) buffering genes, which decrease GC efficacy. Aurora kinase B (AURKB) is overexpressed in resistant ALL in the relapse and is involved in the GC signaling by phosphorylation and suppression of the GR coregulator complex EHMT1/2. AURKB inhibitors potentiate GC sensitivity in B-ALL cell lines and relapsed clinical samples by enhancing GC regulation of effector genes (203).

Pharmacogenetic complex approach, based on three novel methods, was recently suggested by Evans group (85). They combine the polygenomic analysis of primary B- and T-ALL cells with an advanced biostatistical method, in order to identify genes, associated with GC resistance. Further on, they undertook a genomewide CRISP-knockout screening in human ALL cell lines, to prioritize genes, which determine GC resistance. This integrated approach corroborated a polygenomic character of GC resistance. Numerous previously known genes and pathways were confirmed, namely, those involved in B cell development, BCR and IL7R signaling, apoptosis, drugs transport, and inflammation. But, in addition, 14 previously not tagged genes, underlying GC resistance, were identified. Among these is *CELSR2*, which is suppressed in GC resistant samples, possessing also a lower *NR3C1* and a higher *BCL2* expression. A novel resistance mechanism was suggested, where the CELSR2 protein, as a mediator of a non-canonical Wnt signaling (204), positively controls the *NR3C1* and negatively the *BCL2*. Based on these findings, a combined treatment with PRD and Bcl-2 antagonist venetoclax was proposed and successfully validated on *CELSR2* knock-down leukemia cells and xenografted models.

The whole genome sequencing on paired diagnostic and remission T-ALL samples revealed mutations, associated with a resistance to different therapeutics. In particular, IL7R, JAK1, NRAS, and AKT abnormalities are related to the GC resistance, without affecting the sensitivity to vincristine or L-asparaginase (205). Subsequent functional studies revealed that GC resistance was associated with MEK-ERK and AKT/mTOR axes, and upregulation of the pro-apoptotic MCL1 and BclXL.

## Re-Purposed Drugs Can Help To Overcome GC- and Chemoresistance In All

Metabolic upregulation and apoptosis resistance represent convergent points for various signaling pathways, involved in GC resistance in ALL (chapter 2). Consequently, in this section we will introduce the compounds that target precisely these mechanisms, with the focus on the agents already approved by the Food and Drug Administration (FDA) for treatments of some types of cancer or other diseases that demonstrate promising results in preclinical models of the GC-resistant ALL. These drugs may be divided into two groups: (1) drugs with a novel mechanism of cytotoxicity in ALL, which was not considered at the initial approval; (2) drugs that demonstrate the classical mechanism of cytotoxicity in ALL. The data are summarized in the Table 2 and drugs effects are shown in the Figure 2.

**Table 2:** Candidates for drug repurposing against the GC-resistant ALL.

**Compoundand original mechanism**	**Original indications**	**ALL Model**	**Mechanism/ Effects in pre-clinical experiments with ALL**	**Considerations**	References Clinical trials identification number^*^
**2.A. Repurposed drugs with novel mechanisms described in ALL**					
**Cannabidiol** The mechanism is uncertain or there are multiple mechanisms. Different receptors/ targets were proposed as candidates: cannabinoid receptors CB1/CB2; orphan receptors GPR55, serotonin 5-HT_1A_ receptors, μ– and σ– opioid receptors, some ion channels.	FDA approved for treatments of Lennox-Gastaut and Dravet epileptic syndromes: Epidiolex® (oral solution); Sativex® (spray, equal amount of CBD and THC) Arvisol® (oral tablets with pure CBD)	GC-resistant and GC-sensitive continuous T-and B-ALL cell lines	*The novel mechanism*: targets the VDAC channel in the outer mitochondrial membrane, promotes the formation of mPTP and disturbs calcium homeostasis. *Effect*s: enhances autophagy (at low concentrations) and the MPT-related necrosis (at high concentrations), decreases migration.	Routes of administration, vehicle, concentrations, and synergism with other drugs should be considered	(206, 207) *Clinical trials*. As a single agent: NCT02255292 (solid tumors); in a combination with surgery/ radiation: NCT04428203 (prostate cancer); in a combination with chemo- and radiotherapy: NCT03246113, NCT03529448 (glioma); NCT03607643 (gastrointestinal malignancies, glioblastoma multiforme and multiple myeloma).
Ivermectin Milbemycin Moxidectin	FDA approved (Stromectol®) for the treatment of intestinal parasites	B and T lymphoblasts from relapsed patients, cocultured with stromal cells and xenografts	*The novel mechanism*: promotes the intracellular chloride increase and mitochondrial permeabilization *Effects*: induction of the intrinsic apoptosis	Synergism with BH3 mimetics and GCs	(208)
**Mebendazole** Benzimidazole anthelminticagent; binds to the β-tubulin and inhibits cell proliferation.	FDA approved (Vermox™) for the treatment of gastrointestinal worm infections	GC-resistant and GC-sensitive T cell lines.	*The novel mechanism:* promote Notch1 and Hes1 suppression. *Effects:* enhances the GR autoregulation.	Relatively safe even at high doses, effective in nM concentrations, can be administrated by via oral	(73, 209) *Clinical trials* NCT03925662 (colon cancer); NCT02644291 (brain tumors); NCT03628079, (gastric cancer); NCT01729260, NCT01837862 (glioma); NCT02201381 (different cancers)
**Niclosamide**	FDA approved (Niclocide ®) for the treatment of tapeworm infections	GC-resistant and GC-sensitive T cell lines (CCRF-CEM, CEM/ADR5000).	*The novel mechanism*: binds to the glutathione synthetase and limits the NFAT expression. *Effects:* ROS accumulation, decreases the proliferation, interleukin production.	Effective doses are achievable and safe	(210) *Clinical trials* NCT03123978, NCT02807805, NCT02532114 (prostatic cancer); NCT02687009, (colon cancer); NCT02519582 (cancer colorectal); NCT04296851 (adenomous polyposis)
**Tamoxifen** A non-steroid competitive antagonist of nuclear estrogen receptors.	FDA approved (Nolvadex®) for the treatment of metastatic ER-positive breast cancer	GC-resistant cell line (Jurkat)	*The novel mechanism*: binding to the GPER and “off-target” effects *Effects*: causes the autophagy and reverses the sensitivity to GC (non-toxic concentrations); decreases proliferation, causes cell death (at high concentrations)	Effective doses, the protocol for application in pediatric patients	(166) *Clinical trials* NCT00108069 (glioma); NCT00256230, NCT00492505 (melanoma); NCT00710970; NCT02197897 (bladder cancer).
**Tigecycline** The glycylcycline, a broad spectrum tetracycline antibiotic derivative; binds to the bacterial/organellar ribosome and suppresses the protein synthesis	FDA approved (Tygacil®) for the treatment of complicated skin and skin structure infections, complicated intra-abdominal infections and the community-acquired bacterial pneumonia in adults.	GC-sensitive and GC-resistant T-ALL cell lines Primary samples derived from ALL patients. Xenograft mouse models	*The novel mechanism*: inhibits mitochondrial respiration, causing the energy crisis, oxidative stress, and apoptotic cell death. A synergistic effect with doxorubicine and vincristine	Effective doses	(211–213) *Clinical trials* NCT01332786 (R/R AML)
**2.B. Candidate drugs suggested to be extended for therapeutic application in ALL**					
**Azacitidine** **5-Azacytidine** An hypomethylating agent	FDA approved (Vidaza®) for treatment of the myelodysplastic syndrome. *The mechanism*: a hypomethylating agent.	The ALL-7R cell line: GC-resistant, GR-positive	*The mechanism*: a conventional hypomethylating agent. *Effects*: decreases the DNA methylation in the BIM region, increases BIM expression, and reverts the GC resistance.	A toxicity due to an unspecific action and activation of multiple genes; caspase 1 activation, and GR cleavage	(48, 214–216) *Clinical trials* NCT02828358; NCT01861002 (relapsed/ refractory ALL)
**BEZ235** **NVP-BEZ235** **Dactolisib;** A dual inhibitor of the class I PI3K and mTOR kinases by capturing their ATP-binding sites.	Phase I Study in adult R/R ALL patients. Phase Ib study in patients with advanced renal cell carcinoma: an early termination due to the toxicity and a lack of clinical efficacy.	GC-resistant and GC-sensitive continuous T- and B-ALL cell lines. Primary T- and B-ALL cells co-cultured with hBM HS5 stromal cells. Systemic *in vivo* models of T-ALL (including a patient-derived xenograft).	*The mechanism*: a conventional dual inhibition of class I PI3K and mTOR kinases by capturing their ATP-binding sites. *Effects*: enhances the dexamethasone-induced apoptosis in ALL cells (preferentially T-ALL); down regulates Mcl-1 and increases BIM expression; enhances DEX efficiency in T-ALL xenograft models (the tumor load and burden decreases, the EFS increases).	Toxicity	(190, 217–224) *Clinical trials* Next clinical trials were closed due drug toxicity: NCT01453595, NCT01658436, NCT01717898. No one clinical trial registered at the Clinical.trials.gov page provided results about drug safety.
**Venetoclax (ABT-199/GDC-0199)** A specific Bcl-2 protein suppressor	FDA approved (Venclexta®) for adult chronic lymphocytic leukemia and small lymphocytic leukemia	GC-resistant and GC-sensitive continuous T- ALL cell lines Primary R/R and ETP ALL	*The mechanism*: a conventional, specific Bcl-2 protein suppressor. *Effect*s: the mitochondria-dependent apoptosis	Effectiveness in ALL with the upregulation of multiple pro-survival Bcl-2 proteins; safety	(225–228) *Clinical trials* in R/R ALL: NCT03181126, NCT03808610, NCT03504644, NCT03576547, NCT03319901

* *ClinicalTrials.gov, https://clinicaltrials.gov*.

*EFS, the event free survival*.

*ER, estrogen receptors*.

*ETP ALL, ALL of early T cell precursors*.

*GPER, G protein coupled estrogen receptors*.

*MPT, the mitochondrial permeability transition*.

*R/R ALL, Relapsed/ Refractory ALL*.

*R/R AML, Relapsed/ Refractory acute myeloid leukemia*.

*VDAC, the voltage-dependent anion channel*.

### Re-purposed Drugs With a Novel Mechanism in ALL

#### Antibiotic Tigecycline Can Efficiently Control Infections and Kill Leukemic Cells by Targeting Mitochondria

Tigecycline (TGC) is the first commercially available glycylcicline, belonging to a new class of antibiotics, derived from tetracycline (211). TGC binds the bacterial 30S ribosomal subunit and inhibits the bacterial protein translation. It is extremely effective against a broad spectrum of gram-positive and gram-negative pathogens, including the multidrug-resistant ones. Due to similarities between bacterial and mitochondrial ribosomes, TGC is able to suppress the synthesis of mitochondria-encoded proteins, required for OXPHOS, and is efficient in a suppression of some cancer types (212, 229–234). At the same time, TGC exhibits a low toxicity for healthy tissues (229, 233, 234). In addition to the effect on mitochondrial function, TGC inhibits the Wnt signaling and induces autophagy in cervical and gastric cancers (230, 231). Remarkably, TGC is especially effective against therapy-resistant chronic myeloid leukemia stem cells: it inhibits OXPHOS and proliferation and increases their sensitivity to antileukemic drugs (212). As it was discussed in the previous chapter (see section Metabolic Re-programming and Upregulation of the PI3K/Akt/mTOR Pathway Is Related to GC Resistance in ALL), an increased OXPHOS level is a hallmark of GC resistance in ALL. Thus, a possibility of TGC use in therapeutic protocols against ALL is worth to be explored. Up to now, a single pre-clinical study of TGC cytotoxicity against ALL is reported (213). They demonstrated that TGC inhibited mitochondrial respiration, effectively triggered apoptosis and acted synergistically with standard chemotherapeutic drugs vincristine and doxorubicin in multiple GC sensitive and GC resistant ALL cell lines. TGC is also efficient against both newly diagnosed and treatment-refractory clinical samples. Importantly, TGC causes less cytotoxicity in normal hematopoietic cells from leukemia patients. Considering the TGC effectiveness against life-threatening bacterial and fungi infection as well as its good tolerance in ALL patients, including children (235, 236), one may presume that TGC may have a dual function in antileukemic protocols, by targeting heterogeneous populations of leukemic cells, perhaps even primitive leukemia-initiating ones and, at the same time, controlling bacterial and fungal infections.

#### A Multi-Target Drug Tamoxifen May Be Effective Against the GC Resistant ALL

TAM is widely recognized as the gold standard in treatments of the ER positive breast cancer over half a century. However, antiproliferative and cytotoxic effects of TAM against tumor cells of different histogenesis, which do not express classical ERs, including brain and pancreatic cancers, pediatric rhabdoid tumors, melanoma, uterine carcinoma, and T-ALL were reported. Successful *in vitro* experiments and clinical trials represent a solid fundament to reveal the underlying mechanisms and search for new TAM prescriptions as an anticancer drug. TAM easily permeates biological membranes and multiple “non-classical” intracellular TAM targets were reported. TAM suppresses protein kinase C and PI3K/Akt/mTOR pathways and causes a direct suppression of multidrug resistance proteins. In mitochondria, TAM affects membrane fluidity and interacts with pore proteins of the inner membrane, electron transport chain proteins and proteins of the Bcl-2 family. As a result, cell metabolism and proliferation are decreased, and apoptosis is triggered on. TAM also targets lysosomes: it increases the permeability of the lysosomal membrane, causing a release of cathepsine D and activation of autophagy [reviewed in (237)].

Several studies reported a cytotoxic effect of TAM in non-breast cancers, such as melanoma, bladder, and lung ones [reviewed in (238)]. Importantly, TAM shows a synergistic effect with chemotherapeutic drugs, acting via different mechanisms. In particular, TAM enhances the anticancer effect of protein phosphatase 2 inhibitors in pancreatic cancer cell lines through the inhibition of the protein kinase C (239). TAM also enhances the therapeutic effect of a nucleoside analog gemcitabine in the cholangiocarcinoma (240). In the metastatic malignant melanoma, treatment with TAM in a combination with an alkylating agent dacarbazine is more successful than with dacarbazine alone (241). In rhabdoid tumor cells, pan-inhibitor of cyclin-dependent kinases flavopiridol inhibits tumor growth more efficiently, when it is combined with TAM (242).

As it was discussed previously, a GC resistant phenotype in ALL possesses efficient mechanisms for a rapid adaptation to glycolysis inhibition, caused by GCs, by a switch to mitochondrial OXPHOS, with an up-regulation of both glycolysis and mitochondrial metabolism (see section Metabolic Re-programming and Upregulation of the PI3K/Akt/mTOR Pathway Is Related to GC Resistance in ALL). Additionally, autophagy is involved in this switch, but an excessive autophagy observed in a GC-sensitive phenotype is related to a subsequent cell death (see section Effects of GCs on Sensitive Lymphocytes). Thus, TAM, which targets mitochondria and lysosomes and efficiently provokes autophagy, may represent a favorable candidate for ALL treatments.

In our hands, TAM causes mitochondrial dysfunction and autophagy, induces cell cycle arrest and reduces cell viability in GC-resistant Jurkat cells. Autophagy is triggered through the novel membrane G protein-coupled estrogen receptor, GPER. Remarkably, being added in sub-toxic concentrations, TAM partially reverses GC resistance. Healthy lymphocytes are less sensitive to TAM treatment (166).

Although TAM treatment may cause a rapid decrease of the BM cellularity, it shows only a minor effect on a steady state hematopoiesis (243). As TAM has a long history in its clinical use and now proved to exert the antileukemic activity, it may be considered as an appropriate repurposed drug for ALL treatments. But, the application of TAM to pediatric patients requires a more careful consideration.

Several clinical trials, which evaluate the safety and efficacy of TAM for different tumors were successfully undertaken or are in course (Table 2).

#### Cannabidiol Targets Mitochondria

A non-intoxicating cannabinoid cannabidiol (CBD) has a long-term safety and treatment efficacy in pediatric and adult patients with treatment-resistant epilepsies (206). Accordingly, it has been recently approved by FDA for treatments of Lennox-Gastaut and Dravet syndromes [(244); Table 2]. For a long time, CBD was considered as a palliative agent, to improve negative effects of the anticancer therapy, such as pain, nausea, and appetite loss (245–247). At the same time, antineoplastic properties of cannabinoids have been also reported in numerous experimental cancer models (248, 249). In contrast to tetrahydrocannabiol, CBD shows a low affinity for classical cannabinoid receptors CB1 and CB2 and has no undesirable effects on CNS (250). Consequently, its use in anticancer protocols is widely discussed (248–251). On the other hand, the mechanism of CBD cytotoxicity is uncertain. Due to its high lipophilicity, CBD can readily permeate biologic membranes and therefore targets both surface and intracellular structures. Among putative CBD molecular targets some members of the TRP channels family, the orphan cannabinoid receptor GPR55 and mitochondrial VDAC channel have been suggested (207, 248, 251).

Importantly, VDAC acts as a main gatekeeper in the outer mitochondrial membrane that mediates exchange of principal metabolites and ions between mitochondria and cytosol [(143), discussed in Metabolic Re-programming and Upregulation of the PI3K/Akt/mTOR Pathway Is Related to GC Resistance in ALL]. It may adopt different substates, e.g., the completely open one, favoring the transport of metabolites, or the “closed state,” facilitating the mitochondrial Ca^2+^ uptake and preventing the ATP export. A moderate increase of intramitochondrial Ca^2+^ is optimal for the TCA enzymes. Therefore, VDAC exerts the coordination between the aerobic glycolysis in cytosol and OXPHOS in mitochondria, ensuring the metabolic plasticity of a cancer cell. Additionally, VDAC interacts with Bcl-2 family proteins, being involved also in the maintenance of the apoptosis-resistant status. As it was mentioned previously, an upregulation of the aerobic glycolysis and OXPHOS as well as an unpaired apoptosis are classical features of the GC-resistant phenotype in ALL (57, 143).

In our recent study we have tested the CBD efficiency against ALL (207). We have demonstrated that CBD suppressed the viability and impaired the migration of leukemic cells, wherein the T-ALL cell lines were significantly more sensitive than the B-ALL ones. In case of the T-ALL cell line Jurkat mitochondria are proved to be a direct CBD target. CBD seems to directly interact with VDAC channel in the outer mitochondrial membrane, favoring its Ca^2+^-permeable configuration. The resulting Ca^2+^ overload promotes the formation of the mitochondrial transition pore (MTP), membrane potential collapse, and cell death via the MTP-driven necrosis. In our experiments, CBD demonstrates a similar efficiency in both GC-sensitive and GC-resistant cell lineages.

Remarkably, cannabinoids (and CBD in particular) were shown to decrease the P-gp expression and to reverse the MDR activity in ALL cell lines (252). They also inhibit the multidrug transporter ABCG2 (253).

Obviously, the vehicles and routes of the CBD administration, which are necessary to reach the effective concentration in a chemotherapeutic protocol, will differ from those used for the epilepsy treatment. In general, cannabinoids possess a low solubility in aqueous solutions and are relatively unstable (sensitive to oxidation, light and temperature) that should be taken into a consideration during the development of formulations for chemotherapeutic protocols. The effectiveness of CBD, encapsulated in polymeric microparticles, was demonstrated recently in the experimental model of breast cancer [(254), and references therein].

Another important issue to be considered should be the combined effect of CBD with the conventional anti-cancer therapy. The effectiveness of the CBD-loaded microparticles as a potent formulation to improve the doxorubicin- and paclitaxel-based chemotherapy was recently reported (254). Similarly, CBD acts synergistically with the TNF-related apoptosis-inducing ligand (TRAIL) and enhanced the effectiveness of the photodynamic therapy against the colorectal cancer in preclinical models (255, 256). The synergism of CBD with temozolomide and radiotherapy was reported against the glioblastoma (257–259). The effect of CBD in a combination with the conventional therapy was also studied in preclinical models of hematological neoplasms. A synergistic effect of CBD with ibrutinib was demonstrated in cell lines of the diffuse large B-cell lymphoma and mantle cell lymphoma (260). Similarly, a synergism with vincristine and vinblastine was reported in studies with T-ALL and myeloid leukemia- derived cell lines (261). Notably, CBD decreases the cardiocytotoxicity of doxorubicin, which is also used in anti-ALL chemotherapy (262, 263). Thus, the inclusion of CBD in existing anti-leukemic protocols may improve the outcome. However, low CBD concentrations stimulate the T-ALL cells proliferation (207). Thus, the issues of tissue distribution, specific targeting, and safety should be also considered. Additionally, the CBD use in infants and pediatric patients needs to be evaluated. There are several clinical trials in course, which evaluate the safety and efficacy of CBD as a single agent and in a combination with chemo- and radiotherapy against different tumors (Table 2).

#### Anthelmintic Compounds Show Antileukemic Activity

Anthelmintics possess a disruptive activity over the parasite's microtubules, altering the parasite vital functions. Several anthelmintics such as flubendazole, albendazole, and niclosamide demonstrate antitumor properties in several cancer types, including resistant leukemias (73, 208–210, 264–266), albeit the affinity of anthelmintics to the mammalian tubulin appears to be weaker than to the helminthic one (265). The anticancer potential of anthelmintics is also evidenced by several clinical trials, studying safety and efficacy of mebendazole and niclosamide against colorectal, gastric, hepatic, and brain tumors (Table 2). The antileukemic activity of anthelmintics seems to rely on diverse mechanisms. Albendazole alters the MAPK signaling, promotes the mitochondrial dysfunction, such as ΔΨm loss, ROS production, cytochrome c (Cyt-c) release, and causes the intrinsic apoptosis (266). Niclosamide limits the antioxidant system and promotes ROS production by glutathione synthetase inhibition and reduces the NFAT signaling, a vital pathway for leukemic progression (210). Mebendazole was found to inhibit T-ALL by decreasing Notch 1 signaling (reviewed in section Notch Activation) and limiting the *NR3C1* repressor HES1 (73). Mebendazole in both GC resistant and sensitive leukemias represses c-Myc, a key regulator of glucose transporters and cell metabolism. Several groups independently reported that mebendazole targets glucose uptake, reduces cell metabolism, and promotes apoptosis (209). Recently, Mezzatesta and colleagues, using B and T cells from relapsed leukemic patients and patient-derived xenografts for *ex-vivo* experiments, screened 2487 FDA-approved compounds (208). Of the tested anthelmintics, three (ivermectin, moxidectin and milbemycin) display a high cytotoxic effect against leukemic blasts with IC50 values in a low micromolar range, independently on the ALL phenotype. Moxidectin exhibits synergistic effects with DEX and ABT-263 (208).

It should be noted that cancer patients, receiving chemotherapy, show an increased vulnerability to parasite infections [reviewed in (267)] so that the usage of anthelmintics may be justified also by this fact.

### Drugs With a Conventional Mechanism in ALL

#### Hypomethylating Agents May Restore the Expression of the Pro-poptotic BIM Protein

Hypo- and hypermethylation can act as a promoter or a repressor of expression of certain genes, which favor the oncogenic phenotype of a certain cancer, e.g., an overexpression of anti-apoptotic genes, conferring the resistance to cell death induced by chemotherapy, or the inactivation of tumor suppressor genes. Indeed, the methylation profile can be helpful for the diagnosis and prognosis of the patient outcome (268). An aberrant DNA hypermethylation, associated with drug resistance and early relapse, was described in hematologic disorders, in particular, in the myelodysplastic syndrome (214).

Hypomethylating agents (HMA) were proposed, therefore, to be included into chemotherapy protocols. The cytotoxic drug azacitidine (5-Azacytidine, 5-AZA) was shown to act at lower concentrations as a DNA methyltransferase inhibitor, which induces a global DNA hypomethylation (215). 5-AZA (Vidaza^TM^) was approved by FDA for treatments of the myelodysplastic syndrome, where it prolongs the time to the leukemia transformation (216). GC resistance in some cases of ALL is determined by the hypermethylation in lymphocyte-specific open regions of DNA, resulting in a decreased accessibility and a prevention of the correct docking of GC-GR complexes with target genes as the pro-apoptotic BIM [(48), discussed in Effects of GCs on Sensitive Lymphocytes)]. In this study, a gradual decrease of the DNA methylation in the *BCL2L11* region was observed in the GC-resistant ALL-7R cell line during 6 days of the exposure to 5-AZA. Importantly, the combined (5-AZA + DEX) treatment significantly increases BIM expression already at 48 h, causes a synergistic cytotoxicity *in vitro*, decreases the bone marrow infiltration and increases the survival in ALL-7R engrafted mice. However, it should be noted, that the demethylating effect of the HMA is unspecific and can lead to the activation of undesirable genes. For example, the administration of HMA can increase the expression of caspase 1, capable to cleave the GR [(116), discussed in GR May Be Cleaved by Upregulated Caspase 1]. Several clinical trials with a participation of patients with relapsed/ refractory ALL are going on, or are concluded, but their results are still awaiting the FDA approval (Table 2).

#### BH3 Mimetics Inhibits the Anti-apoptotic Members of the Bcl-2 Family

The failure of apoptosis is a hallmark of many types of tumors, including ALL. Proteins of the Bcl-2 family represent a complex network in the apoptosis regulation. The apoptosis execution is ensured by the oligomerization of BAK and BAX proteins in the outer mitochondrial membrane, which mediates its permeabilization and a liberation of Cyt-c and other pro-apoptotic factors into the cytosol. In a pro-survival mode, anti-apoptotic members of the Bcl-2 family proteins (Bcl-2, Bcl-XL, Mcl-1, BFL-1/A1, or Bcl-A1) sequester the BAK and BAX, preventing their oligomerization and apoptosis. Apoptotic and stress stimuli differentially activate other Bcl-2 family members, namely, small proteins, possessing only the BH3 domain (“BH3 only” proteins), such as BIM, Bid, Noxa, and Puma, among others. A balance and interactions between pro-survival and pro-apoptotic proteins determines the threshold for the apoptotic response. Based on this idea, synthetic small molecules that structurally mimic “BH3 only” proteins (“BH3 mimetics”) were developed. BH3 mimetics are capable to bind to and inhibit anti-apoptotic proteins and, accordingly, lower the threshold for apoptosis in cancer cells. Multiple BH3 mimetics with a different specificity were developed. For example, venetoclax (ABT-199/GDC-0199) possesses a high selectivity for the Bcl-2 protein, navitoclax (ABT-263) is dual inhibitor of Bcl-2 and Bcl-XL, whereas a broad spectrum obatoclax (GX15-070) and sabutoclax (B1-97C1) efficiently bind to Bcl-2, Bcl-XL, Mcl-1, and A1 with submicromolar IC50 values. At present, only venetoclax is approved by the FDA (Venclexta®) for treatmentd of adult patients with chronic lymphocytic leukemia (CLL) and small lymphocytic leukemia [(269), and references therein].

Serious alterations in the Bcl-2 proteins profile were found in the GC resistant ALL (discussed in Bcl-2 Superfamily). Bcl-2 is upregulated in the highly aggressive early T precursor (ETP) leukemia, which underlies its sensitivity to venetoclax (225). A mature GC resistant phenotype is characterized by the overexpression of different pro-survival members, including Bcl-2, Bcl-XL, Mcl-1 and, in some highly malignant cases, A1 (57, 139, 162, 194). At the same time, the pro-apoptotic “BH-3 only” BIM protein is downregulated due to the hypermethylation of corresponding gene *BCL2L11* [(48), discussed in Bcl-2 Superfamily]. BIM possesses a high binding affinity to and can efficiently antagonize all members of the anti-apoptotic Bcl-2 proteins family. Its down-regulation results in enhanced levels of all of them. Thus, the pharmacologic strategy to restore the apoptosis triggering in a GC resistant phenotype would consist in (a) application of HMA to restore the BIM expression (see section Hypomethylating Agents May Restore the Expression of the Pro-apoptotic BIM Protein); (b) antagonization of the pro-survival Bcl-2 proteins, using the synthetic BH3 mimetics (269), and (c) a combination of both. However, the narrow anti-Bcl-2 spectrum of venetoclax might reduce its efficiency in malignant cells, which express other anti**-**apoptotic Bcl-2 family proteins. Therefore, the use of broad spectrum BH3 mimetics looks more promising. Unfortunately, broad BH3 mimetics may cause severe collateral effects. The Bcl-XL targeting causes the thrombocytopenia. In turn, Mcl-1 plays an important physiologic role in hepatic and cardiac tissues, neurons, and pluripotent stem cells. Thus, for effective BH3 mimetics use in ALL treatment one needs to verify first the therapeutic window and their safe tolerability profile (270).

Venetoclax has shown an activity against primary ETP samples (225). Despite its narrow specificity to the Bcl-2 protein, venetoclax is also effective against T-ALL cell lines (226). Moreover, it demonstrates very promising results in a combination with classical chemotherapy in clinical trials with the refractory/relapsed T-ALL and ETP patients (227, 228). It turns out that venetoclax therapy is safe, with no clinically significant tumor lysis syndrome and no early patients' death. However, a moderate myelosuppression was reported. There are several ongoing clinical trials, evaluating the Bcl-2 inhibition as a therapeutic strategy for relapsed or refractory ALL (Table 2). The combination of HMA with venetoclax was suggested as a safe and most promising strategy in the AML therapy (271). Thus, it may be considered also for ALL treatments.

The pan-active inhibitor obatoclax sensitizes the GC-resistant ALL cell lines to DEX and causes apoptosis, autophagy, and autophagy-dependent necroptosis (163, 164). Similarly, obatoclax efficiently kills leukemic cells, derived from infants, diagnosed with ALL in *in vitro* assays. It promotes multiple death scenarios, including apoptosis, necroptosis, and autophagy. Importantly, obatoclax acts synergistically with conventional drugs, including DEX (272). Several clinical trials, evaluating the obatoclax safety and effectiveness in hematologic malignances, are in course.

#### PI3K/AKT/mTOR Pathway Inhibitors Are Effective Against ALL

The blockade of the PI3K/AKT/mTOR signaling pathway, which is upregulated in different types of tumors, including the GC-resistant ALL (see section Metabolic Re-programming and Upregulation of the PI3K/Akt/mTOR Pathway Is Related to GC Resistance in ALL), is proposed as a rational therapeutic approach (153, 273). Allosteric mTOR1 inhibitors (rapamycin and its analogs, rapalogs) display promising effects in preclinical models of T-ALL (274, 275) and in a combination with GC synergistically decrease the ALL cells viability (276). Such effects were attributed to the capacity of mTOR to regulate the balance between pro- and anti-apoptotic proteins (188). However, mTOR encompasses two distinct complexes, mTORC1 and mTORC2, which differ in their structure, substrate specificity, and function (277, 278). While mTORC1 induces cell growth by affecting the translational regulators S6K1 and 4E-BP1, mTORC2 mediates cell proliferation and survival via the Akt phosphorylation (279, 280). Therefore, rapalogs could hyperactivate the Akt due to feedback loops between mTORC1, PI3K, and Akt (155). Accordingly, the imidazoquinoline derivative NVP-BEZ235, which inhibits class I PI3K as well as mTORC1/mTORC2 kinases by capturing their ATP-binding sites, may be preferable for treatments (281).

In a panel of T-ALL cell lines and patient-derived T lymphoblasts, NVP-BEZ235 causes cell cycle arrest and apoptosis, and, importantly, also synergizes with the first-line chemotherapeutic agents such as cyclophosphamide, cytarabine, and DEX (282). Activation of the PI3K/Akt/mTOR pathway leads to autophagy (283). It is not surprising that NVP-BEZ235 causes the autophagy activation in T-ALL, and, importantly, the NVP-BEZ235-induced autophagy is not protective against apoptosis (282). However, considering that autophagy may play both pro- and anti-tumor functions, this phenomenon should be studied in more detail.

Using B-ALL patient-derived long-term cultures, the effectiveness of dual inhibitors NVP-BEZ235 (dactolisib) and NVP-BGT226 was tested and compared with those of the pan-PI3K inhibitor NVP-BKM120, combined mTORC1/mTORC2 inhibitors Torin1, PP242, KU-0063794, and the allosteric mTORC1 inhibitor RAD001. Dual PI3K/mTOR inhibitors exerted pronounced antiproliferative and pro-apoptotic effects on ALL cells of different genetic subtypes (284). Yet, a rather variable response of different human B-ALL xenografts was observed in the alternative study, where some xenografts responded better to the single mTOR inhibition (285).

A synergistic antileukemic effect of DEX and NVP-BEZ235 was observed in T-ALL, including *in vitro* (continuous cell lines and primary T-ALL) and systemic *in vivo* models (patient-derived xenograft), but not in B-ALL (190, 286). In T-ALL, NVP-BEZ235 and DEX, added simultaneously, are able to increase BIM and decrease Mcl-1 expression (190). However, rapamycin strongly blocks the GR phosphorylation at Ser211, which is required for its translocation to the nucleus (287).

Up to date, many research groups continue to test dual inhibitors NVP-BEZ235 and NVP-BGT226 in experimental cancer models. Phase I clinical trials were undertaken in patients with different cancers (217–223). Beneficial effects were observed in a small group of relapsed ALL patients (223). However, the unacceptable toxicity of the drug was reported by various researchers (272–277, Table 2). Taking into account that the PI3K/AKT/mTOR pathway is a central regulator of so many metabolic functions in healthy cells and tissues, the clinical perspective for its inhibitors is highly questionable (224).

### High-Throughput Drug Screening Reveals GC Sensitizers Against ALL

The high-throughput screening (HTS) is an efficient strategy for drug discovery. Novel class of drugs with a thioimidazoline moiety, capable to sensitize ALL to GC, was revealed recently by this method (78, 288, 289). In particular, the compound J9 in low nontoxic concentrations is able to increase the GR expression (290). Accordingly, the gene expression pattern in GC-resistant cells co-treated with J9 and DEX is similar to that caused by GCs in sensitive cells. In another study, compound GCS-3 significantly increases the BIM enhancer binding to GRs, resulting in upregulation of BIM and downregulation of C-Myc expression (288, 289). Importantly, GCS-3 is effective against GC-resistant and GC-sensitive xenografts of B-ALL, T-ALL (including ETP), and Philadelphia chromosome positive ALL (288, 289). The knowledge of the action mechanism of effective drugs on their molecular targets, approximation of their interaction mechanisms, and consequent HTS of FDA-approved drugs may reveal new repurposed drugs candidates for ALL treatments.

## Conclusions and Future Perspectives

In general, mechanisms responsible for GC resistance can be divided into the two large groups: those associated with a reduced expression of functional GRs and those that are not. Among the latter, attention should be paid to: (a) general metabolic up-regulation, including glycolysis and OXPHOS, and mechanisms of a flexible switch between them; (b) resistance to apoptosis due to a specific pattern of the Bcl-2 family proteins, including upregulation of different pro-survival members and downregulation of pro-apoptotic proteins (mainly of BIM); (c) upregulation of MDR transporters. A decreased level of the GR expression determines GC resistance by itself, but not necessarily the unresponsiveness to other anticancer compounds. These are abnormalities unrelated to changes in the GR expression that link the GC resistance to the resistance to other drugs and an overall poor prognosis. Then the strategy to improve the outcome for the patients with the GC-resistant ALL is to invoke alternative mechanisms, including the use of some repurposed drugs.

One of the strategies already used in the therapy against the AML is a simultaneous application of BH3 mimetics to inhibit the pro-survival Bcl-2 members and HMA to enhance the BIM expression (see sections Hypomethylating Agents May Restore the Expression of the Pro-apoptotic BIM Protein and BH3 Mimetics Inhibits the Anti-Apoptotic Members of the Bcl-2 Family). As a result, the pro-apoptotic balance among Bcl-2 members will be reached, to restore the apoptosis development in a response to chemotherapy. Another strategy may be metabolic inhibition, to lower a threshold for the regulated cell death, different from apoptosis, such as the autophagy-related cell death, necroptosis, and MTP-related necrosis. In this regard, TAM, a traditional drug, used as an ER antagonist for the chemotherapy of breast cancer, may be an option, due to its numerous “off target” anticancer effects. TAM is proposed as an adjuvant in the therapy in different types of cancers and demonstrates promising antileukemic effects in the preclinical model of T-ALL (discussed in A Multi-Target Drug Tamoxifen May Be Effective Against the GC Resistant ALL). CBD is another highly attractive candidate (discussed in Cannabidiol Targets Mitochondria). CBD, which targets mitochondria in ALL, triggers different antileukemic processes, such as inhibition of glycolysis and OXPHOS, mitochondrial damage, and induction of the MTP-related necrosis. In addition, CBD and TAM inhibit MDR proteins and demonstrate a cardioprotective effect. In case of CBD, its reduced bioavailability maybe the problem. However, a synergistic effect, observed with different chemotherapeutic drugs, allows a significant lowering of the CBD effective concentration. Providing, CBD is integrated in conventional chemotherapeutic protocols, it would also improve a general status and life quality of patients, due to its palliative and cardioprotective effects. Yet additional experiments are required to determine the CBD formulation, administration routes, and dosage. The antibiotic TGC also targets mitochondria and causes cytotoxicity in preclinical ALL models. In addition, it demonstrates an extraordinary effectiveness against drug-resistant infections and good tolerance in ALL patients (see section Antibiotic Tigecycline Can Efficiently Control Infections and Kill Leukemic Cells by Targeting Mitochondria). In a conclusion, the use of the BH3 mimetics and HMA agents as well as repositioning of TGC, TAM, CBD and some anthelminthics (see section Anthelmintic Compounds Show Antileukemic Activity) may substantially improve chemotherapeutic protocols for treatment of the GC-resistant ALL in future. It is expected that the list of FDA-approved compounds for anti-ALL treatments will be extended and new repurposed drugs candidates will be revealed by means of the HTS technology.

## Author Contributions

MO-A and OD contributed to the concept and design of the review. MO-A, LT-L, IP and OD wrote the first draft. MO-A and OD composed the tables. MO-A designed the figures. All authors contributed to the manuscript revision, have read and approved the submitted version.

## Conflict of Interest

The authors declare that the research was conducted in the absence of any commercial or financial relationships that could be construed as a potential conflict of interest.

## Abbreviations

AML: Acute Myeloid Leukemia
ALL: Acute Lymphoblastic Leukemia
ATG: Autophagy Related Gene
AURKB: Aurora Kinase B
BCR: B Cell Receptor
B-ALL: B cell Acute Lymphoblastic Leukemia
BM: Bone Marrow
CA4: Carbon Anhydrase 4
CB: Cannabinoid Receptor
CBD: Cannabidiol
CLL: Chronic Lymphocytic Leukemia
CNS: Central Nervous System
CRAC: Calcium Release Calcium Activated Channel
CREB: Cyclic-AMP Responsive Element Binding Protein
DEX: Dexamethasone
ER: Estrogen Receptors
ETP: Early T cell Precursor
GAPDH: Glyceraldehyde-3-Phosphate Dehydrogenase
GC: Glucocorticoids
GLUT: Glucose Transporter
GR: Glucocorticoid Receptors
GRE: Glucocorticoid Response Element
GSI: Gamma Secretase Inhibitor
HIF-1α: Hypoxia-inducible Factor-1 alpha
HMA: Hypomethylating Agents
HTS: High-Throughput Screening
IL: Interleukin
KD: Knockdown
LSO: Lymphocyte-Specific Open Chromatin
LSC: Lymphocyte-Specific Closed Chromatin
MAPK: Mitogen Activated Protein Kinases
MDM2: Murine Double Minute 2
MDR: Multidrug Resistance pump
mPTP: Mitochondrial Permeability Transition Pore
mTOR: Mammalian Target of Rapamycin
OXPHOS: Oxidative Phosphorylation
P-gp: P-glycoprotein
PI3K: Phosphatidylinositol 3-Kinase
PRD: Prednisolone
PRED: Prednisone
PTEN: Phosphatase and Tensin Homolog
SGK1: Serum and Glucocorticoid inducible Kinase 1
TAM: Tamoxifen
TCA: Tricarboxylic Acid
TCR: T Cell Receptor
TGC: Tigecycline
TRAIL: TNF-Related Apoptosis Inducing Ligand
T-ALL: T cell Acute Lymphoblastic Leukemia
VDAC: Voltage Dependent Anion Channel
ΔΨm: Mitochondrial Membrane Potential.
